# Philadelphia Hospital

**Published:** 1844-01-13

**Authors:** 


### CLINICAL LECTURES AND REPORTS.

---

### PHILADELPHIA HOSPITAL.

CLINIC OF THE JEFFERSON MEDICAL COLLEGE.

*December* 23, 1843.

BY PROFESSOR HUSTON.

#### CASE OF CANCRUM ORIS.

The first subject introduced was "cancrum oris," or gangrenous sore mouth of children. Several cases had recently occurred in the Institution, some of which had proved fatal. The lecturer dwelt on the great severity of the disease, and its insidious character—sometimes commencing as a little ulcer on the gum or the cheek, remaining stationary for weeks, and then suddenly enlarging and involving the gums, alveoli, and even the cheek, and running on rapidly to gangrene of the parts, and death of the patient. At other times beginning with a slight sponginess, and separation of the gum around one or more teeth, gradually destroying the periosteum of the tooth, and descending into the structure of the alveolar process of the jaw, ending in complete destruction of the parts.

A child was shown who had just recovered from an attack, under the judicious treatment of the house surgeon. Several of the temporary teeth were gone, and one rudimental tooth, together with a portion of the alveolar process of the upper jaw. The *cause* of the disease was referred to an enfeebled condition of the nutritive powers, such as is observed in children suffering under long continued irritation of the gastro-intestinal canal, and in those of a strumous diathesis. The *treatment* advised was *constitutional* and *local*, viz.: to cleanse the primæ viæ, improve the habit by proper hygienic means, mild tonics and nutritious food. In the early stage of the disease, the ulcers should be touched with the nitrate of silver, but when the teeth are loose, they must be immediately removed, and also any necrosed portion of the jaw, and the parts freely bathed with some powerful astringent, as solution of the sulphate of copper or zinc, nitro-muriatic acid, &c.

#### PUERPERAL FEVER.

The uterus of a woman who had died of puerperal fever was next shown. She had been of a very feeble constitution, was attacked with the disease four days after confinement, and died twenty-four hours after.

The uterus was somewhat softer and more friable in its texture than usual; its peritoneal coat exhibited very unequivocal marks of inflammation; the fallopian tubes and ovaries were swollen and very dark coloured, and appeared to have been the chief seat of the disease. The lecturer referred to the remarks which he had recently made on the subject of puerperal fever, some of the points of which were illustrated by the present specimen.

#### LEUCORRHŒA.

He next called the attention of the class to the subject of leucorrhœa, and pointed out the symptoms, causes, and seat of the disease. He particularly dwelt on the importance of ascertaining whether the discharge proceeds from the vagina or the uterus. Vaginal leucorrhœa rarely interferes with the functions of the uterus; whereas the other form often supersedes the menstrual discharge, and very generally renders the individual sterile during its continuance. Vaginal leucorrhœa may be either acute or chronic. The acute form is readily cured by rest, low diet, and other antiphlogistic means. The chronic or subacute is more tedious and difficult to relieve; not unfrequently it depends upon faulty nutrition. It is best managed by hygienic means, as riding or sailing, change of air and food, tonics, tog ther with a blister to the sacrum, and frequent injection into the vagina of such astringent washes as the solution of sulphate of alumina, copper, or nitrate of silver.

In *uterine* leucorrhœa astringent injections are not only useless but pernicious. The disease requires to be treated by alteratives, as mercury and iodine, counter-irritation, and a strict avoidance of sexual excitement. Occasionally, copaiba has been serviceable.

#### BY PROFESSOR DUNGLISON.

After some remarks on the great number of cases of asthenic pneumonia in the wards at this season, on which the lecturer had dwelt at length on former occasions, he took up the subject of cerebral hemorrhage, and explained the phenomena presented by it; the pathological lesions, and the appropriate treatment; the remarks on the most common seat of the effusion being illustrated by a recent brain, which he dissected and demonstrated before the class.

#### HEMIPLEGIA.

A case of hemiplegia in a female, preceded by all the symptoms usually assigned to *ramollissement* of the brain was exhibited, in which it was inferred, from the deviation of the eyes and other reasons, that the optic lobes or some portion of the optic nerves was pressed upon by the effused blood. Another case of hemiplegia of shorter duration, also in a female, was shown, in which the loss of motive power was almost entire; sensibility being greatly impaired. The lecturer observed, that although general and local bloodletting and revellents might be of essential service, after the shock induced by the hemorrhage had passed away, with the view of preventing farther effusion, and causing the absorption of that which had already taken place, a period soon arrived at which no therapeutical agents were of any avail; and when it became necessary to trust wholly to the recuperative powers; and that as the clot was absorbed and the neurine recovered its powers, motion would be to a certain degree restored; but that in such cases, the neurine rarely attained its full powers. To illustrate this, a man was brought before the class who had been hemiplegic for upwards of two years, had been subjected to every variety of treatment, and yet the loss of power over the muscles of the affected side was almost as great as at the time of the attack.

#### PERFORATED INTESTINE.

After concluding his remarks on cerebral hemorrhage, the morbid appearances presented in a case of perforation of the intestines were demonstrated. The patient—a female—had suffered for some time under diarrhœa, which had yielded to treatment; after this she left the wards, returning to them with profuse diarrhœa a short time before her death; but strong enough to be able to get out of bed and walk across the ward. She was attacked suddenly with symptoms of peritonitis, followed by decomposition of the features, rapid failure of the powers, and death; which led to the inference that perforation had occurred. The intestines were shown to be perforated in various places by ulceration; in one spot the peritoneal coat alone remained to prevent the contents of the bowels from escaping into the cavity of the peritoneum. Fæcal matter had passed through the ulcerations in considerable quantity into the cavity, and occasioned the fatal peritonitis.

The functional phenomena, which lead to the inference that perforation has occurred in any case, were explained.

An appearance presented by the fallopian tubes led to some comments. Near where the tube terminates in the uterus, a black tumour, of the size of a large marble, existed, which, when cut into, was found to be formed by a cyst filled with a dark coloured pigment. It was not clearly determined, whether this cyst was connected with the tube; but it appeared to be so; and it was suggested, whether this might not be a blighted ovum, which had been arrested at this point and given occasion to the alterations in question.

#### PLEUROPNEUMONIA.

The clinic was concluded with the explanation of the history and morbid appearances presented by a case of pleuropneumonia, which had proved fatal in the first stage of consolidation.

